# Preparation, optimization, and characterization of chitosan-coated solid lipid nanoparticles for ocular drug delivery

**DOI:** 10.7555/JBR.32.20160170

**Published:** 2018-03-14

**Authors:** Feng-zhen Wang, Ming-wan Zhang, Dong-sheng Zhang, Yuan Huang, Li Chen, Sun-min Jiang, Kun Shi, Rui Li

**Affiliations:** 1 Department of Pharmacy, the Affiliated Hospital of Xuzhou Medical University, Xuzhou, Jiangsu 221002, China; 2 School of Pharmacy, Nanjing Medical University, Nanjing, Jiangsu 211166, China; 3 Department of Pharmacy, Tongde Hospital of Zhejiang Province, Hangzhou, Zhejiang 310000, China; 4 Department of Colorectal Surgery, the First Affiliated Hospital of Nanjing Medical University, Nanjing, Jiangsu 210029, China; 5 Department of Pharmacy, the Affiliated Wuxi People's Hospital of Nanjing Medical University, Hospital, Wuxi, Jiangsu 214023, China; 6 Department of Pharmacy, Jiangsu University, the Affiliated Zhenjiang First People’s Hospital, Zhenjiang, Jiangsu 212002, China; 7 Department of Pharmacy, the Affiliated Wuxi Maternity and Child Health Care Hospital of Nanjing Medical University, Wuxi, Jiangsu 214000, China; 8 Department of Orthopedichitosan, Xuzhou Central Hospital, Xuzhou, Jiangsu 221009, China.

**Keywords:** solid lipid nanoparticle, orthogonal design, Box-Behnken design, ophthalmic administration, chitosan

## Abstract

The present study aimed to develop and optimize chitosan coated solid lipid nanoparticles (chitosan-SLNs) encapsulated with methazolamide. Chitosan-SLNs were successfully prepared by a modified oil-in-water emulsification-solvent evaporation method with glyceryl monostearate as the solid lipid and phospholipid as the surfactant. Systematic screening of formulation factors was carried out. The optimized formula for preparation was screened by orthogonal design as well as Box-Behnken design with entrapment efficiency, particle size and zeta potential as the indexes. The entrapment efficiency of the optimized formulation (methazolamide-chitosan-SLNs) prepared was (58.5±4.5)%, particle size (247.7±17.3) nm and zeta potential (33.5±3.9) mV. Transmission electron microscopy showed homogeneous spherical particles in the nanometer range. A prolonged methazolamide *in vitro* release profile was obtained in the optimized chitosan-SLNs suspension compared with methazolamide solution. No ocular damages were observed in the susceptibility test on albino rabbits. The results suggest that the combination of orthogonal design and Box-Behnken design is efficient and reliable in the optimization of nanocarriers, and chitosan-SLNs is a potential carrier for ophthalmic administration.

## Introduction

Solid lipid nanoparticles (SLNs) are a novel colloidal drug delivery system with an inner structure based on solid lipids. It derives from oil-in-water (o/w) emulsion with lipids that are solid at ambient temperature. This desirable drug carrier system demonstrates advantages such as good biocompatibility, low toxicity, drug release modulation, and the possibility of established mass production^[1^–^3]^. Our previous studies demonstrated that SLNs incorporating methazolamide could desirably decrease the intraocular pressure (IOP) of rabbit eyes upon topical application for glaucoma^[4]^. However, its poor corneal permeability became a major challenge as negatively charged SLNs could hardly interact with negatively charged corneal surface. In order to address the problem, many researchers recommended that the particle surface charge should be turned over from negative to positive^[4^–^7]^.

Chitosan, poly [β-(1-4)-linked-2-amino-2-deoxy-d-glucose], is a natural cationic polysaccharide obtained by chitin deacetylation. It has been extensively investigated in the past decades for its application potentialities in pharmaceutical field due to its unique characteristics, such as non-toxicity, biocompatibility and biodegradability, as well as its favorable mucoadhesiveness and biomembrane permeability^[6^,^8^–^12]^. Therefore, chitosan coating has been reported to endow SLNs with some favorable properties^[6^,^13^–^14]^. This cooperation has been very promising, especially in topical administration for ophthalmic diseases^[15^–^17]^.

During the preparation of chitosan-associated SLNs loaded with methazolamide (methazolamide-chitosan-SLNs), we found that phospholipid or glyceryl monostearate (GMS) amount and chitosan concentration could influence the physicochemical properties of methazolamide-chitosan-SLNs. A systematic investigation of multiple complicate variables on methazolamide-chitosan-SLNs fabrication was a prerequisite to achieve optimum physicochemical characteristics before the application of this favorable drug carrier system.

In order to optimize methazolamide-chitosan-SLNs, orthogonal design and Box-Behnken design were carried out successively. The optimal formulation was characterized, the physicochemical properties (surface morphology, particle size, zeta potential, entrapment efficiency, drug loading, etc.) and *in vitro* release behavior were investigated, and *in vivo* studies were also conducted. The optimized methazolamide-chitosan-SLNs showed great potentials for ophthalmic administration.

## Materials and methods

### Materials

Methazolamide was provided by Aoyi Pollen (Hangzhou, China). Chitosan (molecular weight: 50 kDa, deacetylation degree: 95.41%) was purchased from Jinan Haidebei Marline Bioengineering Co. Ltd. Phospholipids (Lipoid S100) were provided by Lipoid (Ludwigshafen, Germany). Polysorbate 80 (Tween 80) was purchased from Jiujiu Bio Tech. Co. Ltd. (Jiangsu, China). Polyethylene glycol 400 (PEG 400) was supplied by the Dow Chemical Company (Shanghai, China). Deionized water was purified by Hitech-K Flow Water Purification System (Hitech Instruments Co. Ltd., Shanghai, China). The osmotic pressure was determined with FM-9X freezing-point osmometer (Instrumental Factory of Shanghai Medical University, China) and pH was measured with PHS-3C precise pH instrument (Shanghai precision & Scientific Instrument Co. Ltd., China). All the other chemicals and reagents were of or above analytical grade.

### Animals

The animal experiments were performed in accordance with the guidelines of the Association for Research in Vision and Ophthalmology (ARVO) statement for the use of animals in ophthalmic and vision research. The studies were conducted in full compliance with ethical principles for laboratory animal care and approved by the local ethics and animal care committee. New Zealand albino rabbits weighing 2.5–3.0 kg were used. All the animals were housed in individual cages with free access to standard food and drinking water, and maintained under standard laboratory conditions with a 12/12 hours light/dark cycle in an air-conditioned room [(25±0.5)°C].

### Preparation of methazolamide-chitosan-SLNs

SLNs loaded with methazolamide were prepared based on a modified emulsification-solvent evaporation and low temperature-solidification^[4]^ with chitosan coating. Briefly, methazolamide, GMS and phospholipids were dispersed in 5 mL ethanol under 70°C water bath. Then, this organic phase was added drop wise into 15 mL aqueous cosurfactant solution (PEG 400 and Tween 80) under magnetic vigorous stirring at the same temperature. After evaporation of the organic solvent, the mixture was then quickly poured into 25 mL chitosan acetate buffer solution (pH 4), stirred at 1,200 r/minute over water-ice bath for 30 minutes, forming methazolamide-chitosan-SLNs. The suspensions obtained were sterilized by filtration through a millipore filter with 0.22 μm pore size.

### Optimization of methazolamide-chitosan-SLNs

#### Orthogonal design

A five-factor, four-level orthogonal design L16(4)^5^ (***Table 1*** and ***2***) was developed to explore the optimum levels of the independent variables including methazolamide amount, phospholipid amount, GMS amount, co-emulsifier concentration and chitosan concentration. To further simplify data processing, entrapment efficiency (EE), drug loading (DL), particle size and zeta potential were respectively and subjectively scored based on the criteria (***Table 2***) and subsequently apportioned with weights of 20%, 10%, 30% and 40%. The weighted sum (20% × EE+ 10% × DL+ 30% × Diameter+ 40% × Zeta) was selected as an overall assessment criterion for methazolamide-chitosan-SLNs optimization.

**Tab.1:** Factors and levels of orthogonal design

Factors	A MTZ (mg)	B Phospholipids (mg)	C GMS (mg)	D Coemulsifiers (%)	E Chitosan (mg/mL)
Level 1	5	0	50	1.0	1.5
Level 2	15	50	100	1.5	2.0
Level 3	25	100	150	2.0	2.5
Level 4	35	150	200	3.0	3.0

MTZ: methazolamide; GMS: glyceryl monostearate.

**Tab.2:** Factors, levels and scoring standard of orthogonal design

EE(100%)	score	DL (%)	score	Diameter (nm)	score	Zeta potential (mV)	score
EE(100%)	score	DL (%)	score	Diameter (nm)	score	Zeta potential (mV)	score
40	1	2	1	250	10	10	1
40,45	2	[2,3)	2	250,275	9	10,11.875	2
45,50	3	[3,4)	3	275,300	8	11.875,13.75	3
50,55	4	[4,5)	4	300,325	7	13.75,15.625	4
55,60	5	[5,6)	5	325,350	6	15.625,17.5	5
60,65	6	[6,7)	6	350,375	5	17.5,19.375	6
65,70	7	[7,8)	7	375,400	4	19.375,21.25	7
70,75	8	[8,9)	8	400,425	3	21.25,23.125	8
75,80	9	[9,10)	9	425,450	2	23.125,25	9
≥80	10	≥10	10	≥450	1	≥25	10

EE: entrapment efficiency; DL: drug loading.

#### Box-Behnken design

Based on our previous studies, a Box-Behnken method was conducted with a 17-run, 3-factor, 3-level design. Three selected independent factors (GMS amount, phospholipid amount and chitosan concentration) were studied at three different levels coded as-1 (low), 0 (medium) and 1 (high). The physicochemical properties of the produced nanoparticles (EE, particle size and zeta potential) were selected as dependent variables. The effect of the independent factors on the dependent variables was represented by a polynomial equation as follows:

(1) Y=b_0_+b_1_A+b_2_B+b_3_C+b_12_AB+b_13_AC_1_+b_23_BC+b_11_AA+b_22_BB+b_33_CC

where Y was the measured response associated with each factor level combination; b_0_ was the arithmetic mean response; b_1_ to b_33_ were the coefficients calculated from the observed experimental values of Y; A, B and C were the coded levels of independent variables. AB, AC, BC and XX (X= A, B, C) represented the interaction and quadratic terms^[10^,^18^–^19]^.

### Characterization of chitosan-SLNs

#### Particle size and zeta potential analysis

The mean particle size and polydispersity index of methazolamide-chitosan-SLNs were determined by photon correlation spectroscopy and the zeta potential was analyzed by laser doppler anemometry. Both measurements were made with ZetaPlus Zeta Potential Analyzer (Brookhaven Instruments Corporation, USA). In each case, the measurement was carried out in triplicate (*n*=3).

#### Entrapment efficiency and drug loading

One mL dispersion of methazolamide-chitosan-SLNs diluted in methanol was sonicated, filtrated and then analyzed by a validated HPLC method established by our laboratory^[4]^ to determine the total amount of methazolamide. Meanwhile, an equal volume of methazolamide-chitosan-SLNs dispersion was ultracentrifuged (Sigma-3k30 High Speed Refrigerated Centrifuge, Sigma Aldrich, German) with centrifugal ultrafiltration tubes (Millipore Amicon Ultra-15, MWCO 100 KDa, Ireland). The liquid phase was moved into the sample recovery chamber through filter membrane, analyzed by HPLC and the quantity of free drug was determined. The entrapment efficiency (EE %) and drug loading (DL %) were calculated by the equations as follows:

(2) $$EE\%=\frac{W_{\mathrm{total drug}}–W_{\mathrm{free}\mathrm{drug}}}{W_{\mathrm{total drug}}}\times 100\%$$

(3) $$DL\%=\frac{W_{\mathrm{total drug}}{–W}_{\mathrm{free drug}}}{W_{\mathrm{total drug}}{–W}_{\mathrm{free drug}}+W_{\mathrm{emulsifiers}}+W_{\mathrm{lipid}}}\times 100\%$$

where W_total drug_ was the mass of total methazolamide in methazolamide-chitosan-SLNs; W_free drug_ was the mass of free methazolamide detected in the supernatant after centrifugation; W_emulsifiers_ and W_lipid_ were the mass of emulsifiers and lipid initially used.

#### Transmission electron microscopy (TEM) analysis

The morphology was observed by a transmission electron microscope (JEM-200 CX, JEOL, Tokyo, Japan). Sample of the methazolamide-chitosan-SLNs dispersion was mounted on a carbon-coated copper grid, completely dried under vacuum and then examined^[19]^.

#### Fourier transform infrared spectroscopy (FTIR)

FTIR spectra for methazolamide, methazolamide loaded chitosan-SLNs, blank chitosan-SLNs and physical mixture of methazolamide and blank chitosan-SLNs were monitored using a Fourier Transformation Infrared Spectrophotometer (TENSOR 27, Bruker, Germany). KBr discs of the lyophilized formulations were prepared and analyzed in the wavelength range of 400 cm^–1^–4,000 cm^–1^.

#### Differential scanning calorimetry (DSC)

DSC analyses for methazolamide, methazolamide loaded chitosan-SLNs, blank chitosan-SLNs and physical mixture of methazolamide and blank chitosan-SLNs were performed using a differential scanning calorimeter (DSC 204, Netzsch, Germany). Samples were placed in flat-bottomed aluminum pan and heated at a constant rate of 10 °C/minute in nitrogen in a temperature range of 20 °C–500 °C.

#### Powder X-ray diffractometry (XRD)

Powder X-ray diffraction patterns for methazolamide, methazolamide loaded chitosan-SLNs, blank chitosan-SLNs and physical mixture of methazolamide and blank chitosan-SLNs were obtained by a powder X-ray diffractometer (D8 Advance, Bruker-AXS, Germany). XRD studies were performed on the samples by exposure to CuKα radiation (40 kV, 30 mA) and scanned from 3° to 40°, 2θ at a step size of 1° and step time of 1 minute.

### ***In vitro*** release study

The release behavior of methazolamide from methazolamide-chitosan-SLNs dispersion was investigated by dialysis method in artificial tear fluid (ATF)^[20]^. Dialysis bags (MCWO 12,000–14,000, Sigma, USA) loaded with 2 mL samples were dipped into 100 mL dissolution medium stirred at (37±0.5) °C using a ZRS-8G Drug Dissolution Tester (Tianjin University Radio Factory, China) with paddles rotating at 50 r/minute. At regular time intervals, aliquots (1 mL) were withdrawn from the medium and then replenished with the same volume of fresh medium. The contents of released methazolamide were determined by HPLC^[4]^. All measurements were performed in triplicate (*n*=3).

### Susceptibility test

The osmotic pressure and pH of methazolamide-chitosan-SLNs were within the acceptable range^[21]^. To investigate the acute ocular tolerance of methazolamide-chitosan-SLNs, a drop of the dispersion was applied to one eye of the rabbit while the contralateral eye received the same volume of physiological saline as control. Administration was performed every 30 minutes for 8 hours. The susceptibility was evaluated according to a modified Draize test^[22^–^23]^.

### ***In vivo*** study

To investigate the intraocular pressure (IOP) lowering effect of methazolamide-chitosan-SLNs on rabbits, a randomized, crossover, double-blind, placebo-controlled study was carried out with an animal washout period of one week. The IOP was measured using a standardized YJL Impression Tonometer (MingRen Medical Instrument Co., Ltd. of Suzhou, China) and IOP decrease (% decrease in IOP) as a function of time was plotted to assess the IOP lowering effect.

### Statistical analysis

Statistical analysis of the results was performed using extreme analysis and analysis of variance (ANOVA). The statistical analysis was computed with the SPSS^®^ software. Differences were considered significant when *P * 0.05.

## Results

### Optimization of methazolamide-chitosan-SLNs

#### Orthogonal design

***Table 3*** displays the EE, DL, particle size and zeta potential of the prepared chitosan-SLNs as well as their corresponding scores based on the criteria subjectively prescribed in ***Table 3***. As presented in ***Table 4***, for a specific independent, the K_i_ (i=1, 2, 3 or 4) value is the average of the resulting dependent values under level i, which is used to determine the optimal level for the five independents. The difference between the maximal and minimal values of K_i_ is defined by the range value R. A higher R value indicates a greater effect on the dependent^[24^–^25]^. The influence of the independents on the weighted sum of the dependents is in the following order: C (GMS)>B (phospholipids)>E (chitosan)>D (co-emulsifiers)>A (methazolamide), based on R values (***Table 4***), which suggests that C, B, E are three significant factors. The amount of methazolamide shows the significant and positive effect on DL (*P* 0.05). Therefore, to achieve a high DL, a relatively large amount of methazolamide is expected to be applied.

**Tab.3:** Composition of SLN formulation in the orthogonal design and result of the experiment

Formulation composition effect	1	2	3	4	5	6	7	8	9	10	11	12	13	14	15	16
MTZ (mg)	5	5	5	5	15	15	15	15	25	25	25	25	35	35	35	35
Phospholipids (mg)	0	50	100	150	0	50	100	150	0	50	100	150	0	50	100	150
GMS (mg)	50	100	150	200	100	50	200	150	150	200	50	100	200	150	100	50
Coemulsifiers (%)	1	1.5	2	3	2	3	1	1.5	3	2	1.5	1	1.5	1	3	2
Chitosan (mg/mL)	1.5	2	2.5	3	3	2.5	2	1.5	2	1.5	3	2.5	2.5	3	1.5	2
EE%	29.30	60.80	58.90	56.50	62.40	57.20	48.00	54.00	42.90	41.80	70.00	58.40	24.00	36.50	58.70	76.90
score	1	6	5	5	6	5	3	4	2	2	8	5	1	1	5	9
DL%	1.01	2.05	1.29	0.77	6.59	4.81	2.12	2.16	4.04	1.90	8.09	4.75	3.80	4.61	6.97	10.96
score	1	2	1	1	6	4	2	2	4	1	8	4	3	4	6	10
Diameter	275.2	347.5	433.7	556.1	393.3	294.6	542.8	448.7	268.2	382.5	547.9	325.3	414.0	450.1	354.2	311.6
score	8	6	2	1	4	8	1	2	9	4	1	6	3	1	5	7
Zeta potential	19.3	14.4	13.0	12.0	18.3	19.4	6.4	9.3	10.3	9.4	7.6	14.2	24.6	10.6	6.7	11.6
score	6	4	3	3	6	7	1	1	2	1	1	4	9	2	1	2
Sum	5.1	4.8	2.9	2.6	5.4	6.6	1.5	2.0	4.3	2.1	3.1	4.8	5.0	1.7	3.5	5.7

MTZ: methazolamide; GMS: glyceryl monostearate; EE: entrapment efficiency; DL: drug loading.

**Tab.4:** Statistical analysis of orthogonal design for sum, EE, DL, particle size, and zeta potential of MTZ-CS-SLNs

SLN characterization	Statistical parameter	MTZ	Phospholipids	GMS	Coemulsifiers	Chitosan
Sum	K_1_	3.850	4.950	5.125	3.275	3.175
	K_2_	3.875	3.800	4.625	3.725	4.075
	K_3_	3.575	2.750	2.725	4.025	4.825
	K_4_	3.975	3.775	2.800	4.250	3.200
	R_j_	0.400	2.200^a^	2.400^a^	0.975	1.650^a^
EE	K_1_	51.375	39.650	58.350	43.050	45.950
	K_2_	55.400	49.075	60.075	52.200	57.150
	K_3_	53.275	58.900	48.075	60.000	49.625
	K_4_	49.025	61.450	42.575	53.825	56.350
	R_j_	6.375	21.800^a^	17.500^a^	16.950	11.200
DL	K_1_	1.280	3.860	6.218	3.123	3.010
	K_2_	3.920	3.343	5.090	4.025	4.793
	K_3_	4.695	4.617	3.025	5.185	3.662
	K_4_	6.585	4.660	2.147	4.147	5.015
	Rj	5.305^a^	1.317	4.071	2.062	2.005
Diameter	K_1_	403.125	337.675	357.325	398.350	365.150
	K_2_	419.850	368.675	355.075	439.525	367.525
	K_3_	380.975	469.650	400.175	380.275	366.900
	K_4_	382.475	410.425	473.850	368.275	486.850
	Rj	38.875	131.975^a^	118.775	71.250	121.700^a^
Zeta potential	K_1_	14.675	18.125	14.475	12.625	11.175
	K_2_	13.350	13.450	13.400	13.975	10.675
	K_3_	10.375	8.425	10.800	13.075	17.800
	K_4_	13.375	11.775	13.100	12.100	12.125
	R_j_	4.300	9.700^a^	3.675	1.875	7.125^a^

^a^*P* 0.05. MTZ: methazolamide; GMS: glyceryl monostearate; EE: entrapment efficiency; DL: drug loading.

To further optimize the formulation with the appropriate value of the main influencing factors (C, B and E), a Box-Behnken design was then applied with the EE, particle size and zeta potential as indexes, while the DL was excluded since none of the three factors presented significant effects on DL. The subsequent formulations were prepared with 35 mg methazolamide and 2% co-emulsifier.

#### Box-Behnken design

**Fig.1:**
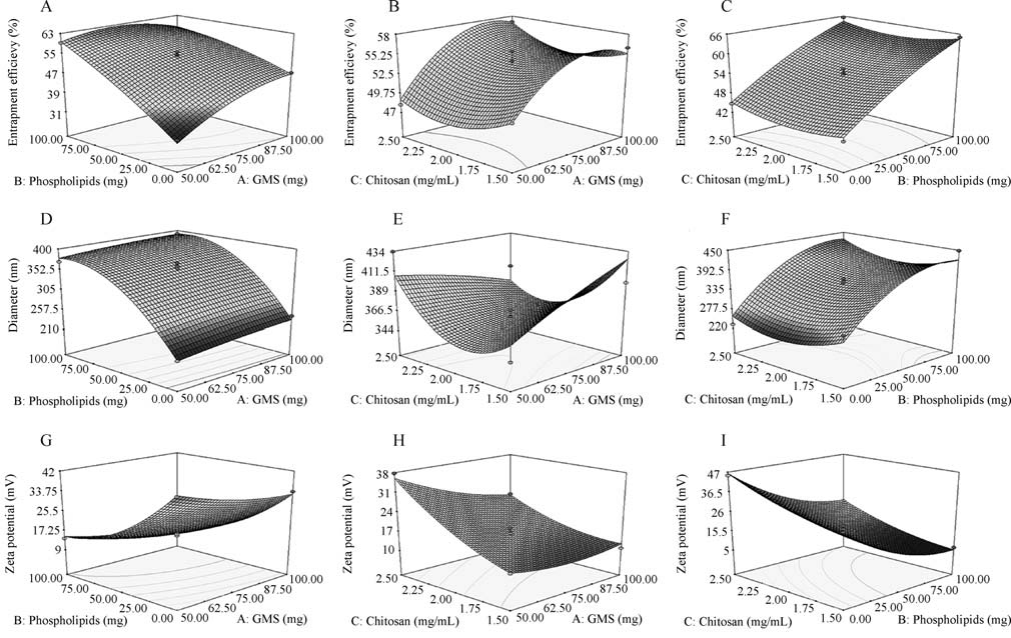
Response surface plots.

The characteristics of the 17 methazolamide-chitosan-SLNs formulations are given in ***Table 5***. The three dimensional response surface plots depicting the effects of two predetermined factors with the third fixed at a constant (middle) level are presented in ***Fig. 1***. Statistical analysis indicated that the observed responses of EE, particle size and zeta potential are all dependent on the selected variables [GMS amount (A) and phospholipid amount (B), chitosan concentration (C)] to some extent. ***Table 6*** shows the regression coefficient values and their corresponding *P*-values. A positive coefficient value in the polynomial equation exhibits a synergistic effect between the independent and dependent, while a negative value indicates an antagonistic effect^[26]^.

**Tab.5:** Observed responses in Box-Behnken experimental design for MTZ-CS-SLNs

	Dependent variables	Independent variables
Run	A: GMS (mg)	B: Phosph-olipids (mg)	C: chitosan (%)	EE%	DL%	Diameter (nm)	Zeta potential(mv)
1	75	100	1.5	65.04	8.68	448.3	6.41
2	75	50	2	53.61	8.86	356.8	16.93
3	75	50	2	53.65	8.75	367.5	16.26
4	75	0	1.5	43.06	8.66	290.3	29.78
5	50	100	2	59.26	9.74	370.7	13.91
6	50	50	2.5	48.11	12.61	433.5	37.7
7	100	0	2	47.2	9.23	242.1	33.6
8	75	50	2	53.55	8.72	359.8	15.66
9	100	100	2	56.88	6.83	391.3	9.78
10	75	50	2	54.33	6.16	350.7	15.08
11	100	50	2.5	57.1	6.63	393.2	22.51
12	50	50	1.5	49.89	9.53	345.7	13.28
13	75	50	2	55.67	4.53	357.1	18.28
14	100	50	1.5	56.14	7.55	399.1	10.89
15	75	100	2.5	65.55	4.11	411.3	19.38
16	75	0	2.5	44.75	5.71	231.5	45.24
17	50	0	2	32.74	13.24	214.9	41.12

**Tab.6:** Statistical analysis for Box-Behnken design of EE, diameter, and zeta potential

Parameters	EE	Diameter	Zeta potential
	Coefficient	*P*-value	Coefficient	*P*-value	Coefficient	*P*-value
Intercept	54.16	0.0001	358.38	0.0015	16.44	0.0001
A-GMS	3.42	0.0001	7.610	0.4179	-3.65	0.0004
B-phospholipids	9.87	0.0001	80.35	0.0001	-12.53	0.0001
C-chitosan	0.17	0.7092	-1.74	0.8498	8.06	0.0001
AB	-4.21	0.0003	-1.65	0.8988	0.85	0.3276
AC	0.69	0.3115	-23.43	0.1032	-3.20	0.0054
BC	-0.30	0.6528	5.45	0.6762	-0.62	0.4648
A^2	-3.47	0.0008	-3.05	0.8095	2.03	0.0364
B^2	-1.68	0.0290	-50.58	0.0043	6.13	0.0001
C^2	2.11	0.0106	37.55	0.0178	2.63	0.0123
R-squared	0.9893		0.9414		0.9916	

^a^*P* 0.05. MTZ: methazolamide; GMS: glyceryl monostearate; EE: entrapment efficiency; DL: drug loading.

The regression Eq. (4) constructed for EE is presented below:

(4) $$Y_{\mathrm{EE\%}}\mathrm{=54}\mathrm{.16+3}\mathrm{.42\times A+9}\mathrm{.87\times B+0}\mathrm{.17\times C}–\mathrm{4.21\times A\times B+0}\mathrm{.69\times A\times C}–\mathrm{0.30}\mathrm{\times B\times C}\mathrm{3.47\times AA}–1\mathrm{.68\times BB}\mathrm{+2.11\times CC}$$

Quantitative estimation indicated that the amount of phospholipids had a prime influence on EE for its relatively large positive coefficient (9.87), suggesting that an increased amount of phospholipids in the formulation leads to an increase in EE. The positive coefficient value of GMS indicates that with the increase of GMS, the amount of methazolamide incorporated in chitosan-SLNs also increases, as the matrix GMS provides great accommodation to encapsulate lipophilic drugs due to its good lipophilicity and high monoglyceride ratio (40%–50%)^[18^,^27]^. Eq. (5) explains the effect of factors on particle size:

(5) $${\mathrm{YD}}_{\mathrm{iameter}}\mathrm{=358}\mathrm{.38+7}\mathrm{.61\times A+80}\mathrm{.35\times B}–1\mathrm{.74\times C}–1\mathrm{.65\times A\times B}–\mathrm{23}\mathrm{.43\times A\times C}\mathrm{+5.45\times B\times C}–3\mathrm{.05\times AA}–\mathrm{50}\mathrm{.58\times BB}\mathrm{+37.55\times CC}$$

***Fig. 1D–F*** shows the response surface plot for particle size in response to the investigated factors. The positive coefficient value of B (phospholipids) suggests an unfavorable effect of phospholipids on nanoparticle size, as the existence of negatively charged phospholipids can increase positively charged chitosan amount on the surface of SLNs, thus increasing the diameter of chitosan-SLNs^[4]^. According to the equation, chitosan concentration in the coating phase has no direct but a significant quadratic influence on the particle size of chitosan-SLNs.

The regression equation for zeta potential in terms of factors is described as follows:

(6) $$Y_{\mathrm{Zeta}}\mathrm{=16}\mathrm{.44}–3\mathrm{.65\times A}–\mathrm{12}\mathrm{.53\times B+8}\mathrm{.06\times C}\mathrm{+0.85\times A\times B-3}\mathrm{.20\times A\times C}–0\mathrm{.62\times B\times C+2}\mathrm{.03\times AA+6}\mathrm{.13\times BB}\mathrm{+2.63\times CC}$$

By analyzing this second order polynomial mode, zeta potential wassignificantly influenced by all the three independent variables. Specifically, it decreased with the increase of the negatively charged phospholipid amount and increases with the increase of positively charged chitosan concentration. Besides, GMS exerted a negative effect on zeta potential, which may be attributed to the slight ionization of fatty acids from GMS to some extent^[27]^. Chitosan coating endowed SLNs with positively charged particle surface due to the protonated amino groups of chitosan, which was highly expected because this can favor the interaction between SLNs and the negatively charged mucous membranes (such as cornea, conjunctiva) and consequently increase the residence time of the associated drug^[23]^.

The result above indicated that the phospholipid amount played a significant role in all the three dependent responses. An increased phospholipid amount led to higher EE, a larger particle size and lower zeta potential. In order to solve this conflict, a proper phospholipid amount must be determined.

Repeatability of the experiments based on Box-Behnken design was examined through five replicates at the center point (middle level) (75 mg GMS, 50 mg phospholipids and 2 mg/mL chitosan solution). The results showed similar dependent response values (***Table 6***), which indicated a good reproducibility for the formulations of methazolamide-chitosan-SLNs.

#### Optimization and validation

The optimized formulation was obtained based on the criteria of maximum EE and maximum zeta potential and minimum particle size. Therefore, a new batch of methazolamide-chitosan-SLNs was prepared to validate the reliability of optimization. The composition of the optimum formulation was accomplished as 100 mg GMS, 20 mg phospholipids and 2.5 mg/mL chitosan with the predicted values as EE 52%, particle size 273 nm, and zeta potential 36 mV. The optimized formulation prepared demonstrated the actual values of EE as (58.5±4.5)%, particle size as (247.7±17.3) nm and zeta potential as (33.5±3.9) mV, which was in good agreement with the predicted values, thus indicating the validity and effectiveness of the Box-Behnken design.

### Characterization of optimized methazolamide-chitosan-SLNs

#### Transmission electron microscopy (TEM) analysis

**Fig.2:**
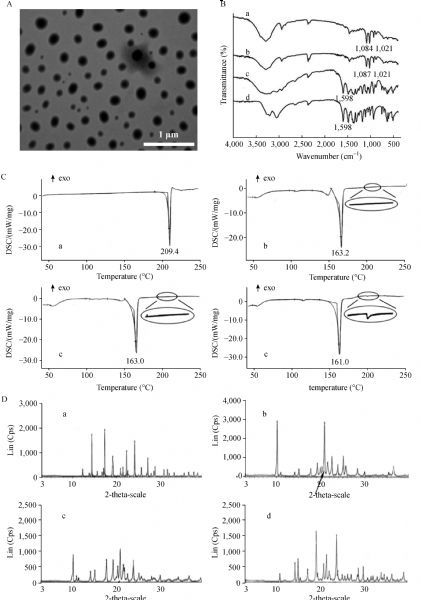
Characterization of methazolamide-chitosan-SLNs.

TEM image of chitosan-SLNs loaded with methazolamide is presented in ***Fig. 2A***. The particles showed spherical morphology with a smooth surface and a narrow size range. The diameter based on TEM (240 nm) was similar to the value determined by photon correlation spectroscopy (about 250 nm).

#### Fourier transform infrared spectroscopy (FT-IR)

***Fig. 2B*** shows the FTIR spectra of blank chitosan-SLNs (a), methazolamide-chitosan-SLNs (b), physical mixture of methazolamide and blank chitosan-SLNs (c) and methazolamide (d). The characteristic peak of methazolamide at 1,598 cm^-1^ of CO-NH was seen in both physical mixture and methazolamide but disappeared in methazolamide-chitosan-SLNs spectrum as methazolamide was expected to be incorporated within the nanoparticle rather than a simple component of a mixture.

#### Differential scanning calorimetry (DSC)

The DSC profiles of methazolamide, methazolamide-chitosan-SLNs, blank chitosan-SLNs and physical mixture of methazolamide and blank chitosan-SLNs are presented in ***Fig. 2C***. Methazolamide exhibits characteristic endothermic peak at 209.4 °C followed by an irregular exothermic peak. The DSC profile of methazolamide-chitosan-SLNs is different from that of physical mixture of methazolamide and blank chitosan-SLNs while similar to chitosan-SLNs, indicating that the methazolamide is successfully loaded in chitosan-SLNs.

#### Powder X-ray diffractometry

X-ray powder diffractograms of methazolamide, methazolamide-chitosan-SLNs, blank chitosan-SLNs and physical mixture of methazolamide and blank chitosan-SLNs are presented in ***Fig. 2D***. The crystalline peaks of methazolamide-chitosan-SLNs do not show the specific sharp crystal peaks of methazolamide and are different from that of the physical mixture, indicating that methazolamide is completely and successfully encapsulated into the core of chitosan-SLNs.

### ***In vitro*** release study

**Fig.3:**
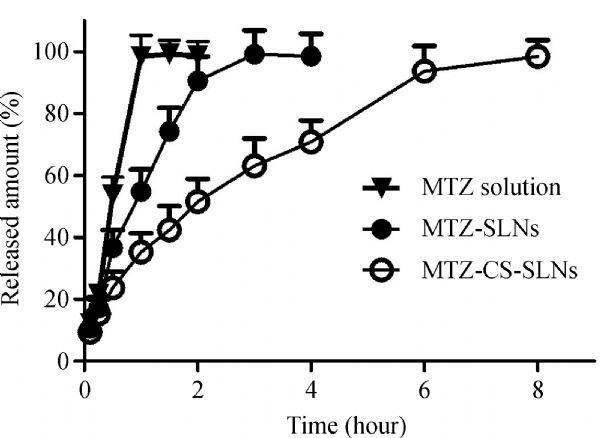
***In vitro*** release profile of the methazolamide solution, methazolamide-SLNs and methazolamide-chitosan-SLNs.

The cumulative methazolamide release (%) as a function of time (hour) is shown in ***Fig. 3***. methazolamide solution, methazolamide-SLNs and methazolamide-chitosan-SLNs containing the same concentration of methazolamide [about 0.5% (w/v)] were in good sink conditions for the *in vitro* release study. methazolamide solution and methazolamide-SLNs revealed a fast release of methazolamide in the first hour and about 100% of methazolamide is released in the fourth hour, respectively. methazolamide-chitosan-SLNs dispersion exhibited a biphasic release profile: an initial burst release about 50% of methazolamide within the first two hours followed by a gradual and sustained release in the following 6 hours. Compared with methazolamide solution and methazolamide-SLNs, methazolamide-chitosan-SLNs displayed a better drug release profile^[7^,^28]^.

### Susceptibility test

**Fig.4:**
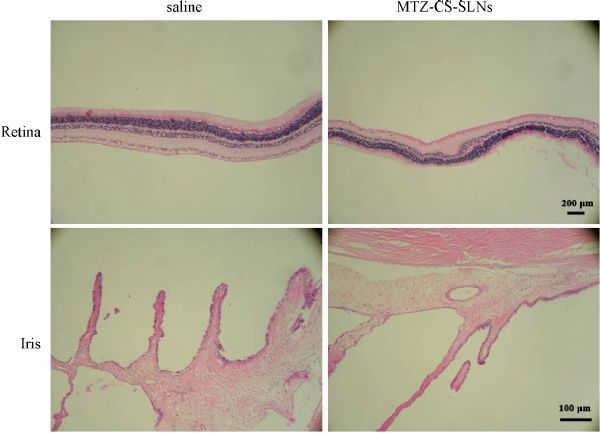
Representative images of haematoxylin and eosin (H&E) -stained iris and retina of rabbit eyes after drug application.

Ophthalmic irritation, a common drawback in ocular drug development, often restricts the drug from clinical use. Rabbits with normal ocular surface structures were selected as animal models. The ocular condition was observed after each application. No macroscopic manifestations or clinically abnormal signs, such as corneal opacity, iris hyperaemia or redness, conjunctiva swelling or discharge, were observed in any chitosan-SLNs-exposed eyes or control eyes (***Fig. 4***). The scores according to the Draize method are zero. It is reasonable to conclude that the chitosan-SLNs carrier was well tolerated in rabbit eyes.

### ***In vivo*** studies

**Fig.5:**
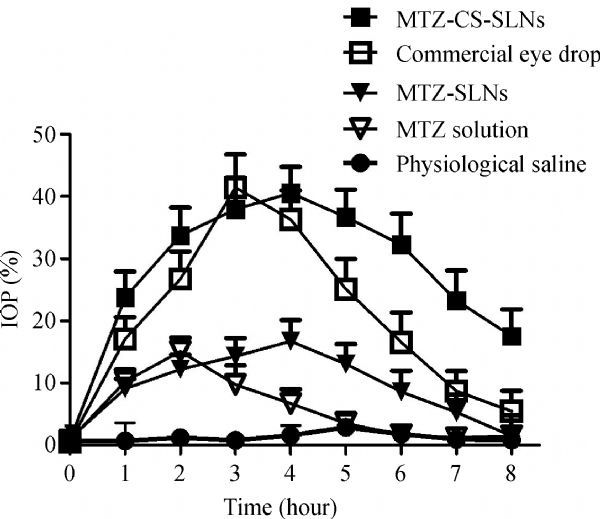
Percentage decrease in intraocular pressure (IOP) after administration of methazolamide solution, methazolamide-SLNs, methazolamide-chitosan-SLNs, commercial eye drop and physical saline solution.

***Fig. 5*** demonstrates the IOP percentage decrease (ΔIOP) versus time profiles of methazolamide loaded chitosan-SLNs formulation, the commercial brinzolamide Eye Drop (AZOPT), methazolamide solution, methazolamide loaded SLNs formulation and physical saline solution. The area under the percentage decrease in IOP-time curve (AUC 0–8 hours) of methazolamide-chitosan-SLNs, commercial eye drop, methazolamide-SLNs and methazolamide solution are 237.8, 175.2, 81.2 and 49.9 mmHg × hour, respectively. The data demonstrated that methazolamide-chitosan-SLNs had a good effect on ΔIOP, which was significantly better than methazolamide solution (*P* 0.05) and relatively better than AZOPT. The efficacy may be ascribed to the favorable properties of the carrier (chitosan-SLNs), such as mucoadhesiveness, positively charged surface, and biomembrane permeability. At physiological pH, the corneal epithelium is negatively charged (the isoelectric point is 3.2), and easy to interact with positively charged chitosan-SLNs, which decreases its tear wash-out rate, prolongs residence time and favors its paracellular permeability^[29]^. Besides, chitosan has been reported to reversibly disrupt corneal epithelial tight junctions^[14^,^30]^, which can also lead to the improved biomembrane permeability of chitosan-SLNs.

## Discussion

Methazolamide is a systemically administered carbonic anhydrase inhibitor for glaucoma treatment, however, the carbonic anhydrase enzyme in many organs and tissues often leads to systemic side effects. Though direct topical administration to the eye can reduce side effects, the application of methazolamide suffers from challenges due to its low aqueous solubility and poor corneal permeability^[31]^. Fortunately, many studies have proved that solid lipid nanoparticles(SLNs) is a suitable carrier to improve ocular drug delivery. With their lipophilic character, small size and particulate nature^[5]^, SLNs can adhere to ocular membranes and prevent tear wash-out^[32]^.

Our previous studies have indicated that the encapsulation of methazolamide to SLNs is an alternative ocular drug delivery system, which can decrease the IOP of rabbit eyes^[4]^. However, conventional SLNs often show negative charge, making it difficult to interact with the negatively charged cornea surface. In order to increase the corneal permeability, a turnover of particle surface charge from negative to positive is recommended^[33]^. Chitosan, a natural cationic polysaccharide, has advantages including favorable mucoadhesiveness, biomembrane permeability and low toxicity^[34]^. Thus, the cooperation of chitosan and SLNs can improve drug bioavailability by enhancing penetration across mucosal barriers and prolonging residence time in the absorption region.

In this study, the ocular drug delivery system methazolamide-chitosan-SLNs was successfully prepared and optimized. The influence factors (such as the amount of methazolamide, phospholipids or GMS and the concentration of coemulsifiers or chitosan) of the nanoparticle formulations were optimized based on an orthogonal design and a Box-Behnken design with particle size, zeta potential, EE and DL as indexes. The optimized formulation was based on 100 mg GMS, 20 mg phospholipid and a coating phase of 2.5 mg/mL chitosan acetate solution with (58.5±4.5)% EE, (247.7±17.3) nm particle size and (33.5±3.9) mV zeta potential. All the indexes were in good agreement with the values anticipated by the Box-Behnken design. The combination of the two optimizing methods is efficient and reliable. *In vivo* results showed that methazolamide-chitosan-SLNs were successful in ocular delivery of methazolamide, with a marked decrease in IOP and better sustainability than methazolamide-SLNs, indicating that methazolamide-chitosan-SLNs could have favorable properties and potentiality for the treatment of local ophthalmic diseases.

In conclusion, the present study has revealed that chitosan coated SLNs can successfully deliver methazolamide in glaucoma treatment. However, the exact mechanism of chitosan in promoting permeation in ocular region is not clear. Therefore, we will focus on the transport route of methazolamide-chitosan-SLNs by ocular administration in future studies
